# Urinary titin N-fragment as a predictor of decreased skeletal muscle mass in patients with interstitial lung diseases

**DOI:** 10.1038/s41598-023-36827-5

**Published:** 2023-06-15

**Authors:** Masatoshi Hanada, Yuji Ishimatsu, Noriho Sakamoto, Yoshiko Akiyama, Takashi Kido, Hiroshi Ishimoto, Masato Oikawa, Hiroki Nagura, Rina Takeuchi, Shuntaro Sato, Hideaki Takahata, Hiroshi Mukae, Ryo Kozu

**Affiliations:** 1 Department of Rehabilitation Medicine, Nagasaki University Hospital, Nagasaki, Japan; 2 Department of Physical Therapy Science, Nagasaki University Graduate School of Biomedical Sciences, Nagasaki, Japan; 3 Department of Nursing, Nagasaki University Graduate School of Biomedical Sciences, 1-7-1 Sakamoto, Nagasaki, 852-8520 Japan; 4 Department of Respiratory Medicine, Nagasaki University Graduate School of Biomedical Sciences, Nagasaki, Japan; 5 Clinical Research Center, Nagasaki University Hospital, Nagasaki, Japan

**Keywords:** Diseases, Health care, Medical research

## Abstract

This study aimed to examine the validity of urinary N-terminal titin fragment/creatinine (urinary N-titin/Cr) reflecting muscle damage biomarker in patients with interstitial lung disease. This retrospective study enrolled patients with interstitial lung disease. We measured urinary N-titin/Cr. Furthermore, we measured the cross-sectional areas of the pectoralis muscles above the aortic arch (PM_CSA_) and erector spinae muscles of the 12th thoracic vertebra muscles (ESM_CSA_) to assess muscle mass until 1 year. We examined the correlation between urinary N-titin/Cr and the change in muscle mass. We plotted receiver operating characteristic curves to estimate the cut-off points for urinary N-titin/Cr for distinguishing the greater-than-median and smaller-than-median reduction of muscle mass after 1 year. We enrolled 68 patients with interstitial lung disease. The median urinary N-titin/Cr value was 7.0 pmol/mg/dL. We observed significant negative correlations between urinary N-titin/Cr and changes in the PM_CSA_ after 1 year (p < 0.001) and changes in the ESM_CSA_ after 6 months (p < 0.001) and 1 year (p < 0.001). The cut-off points for urinary N-titin/Cr were 5.2 pmol/mg/dL and 10.4 pmol/mg/dL in the PM_CSA_ and ESM_CSA_, respectively. In summary, urinary N-titin/Cr may predict muscle loss in the long-term and act as a clinically useful biomarker reflecting muscle damage.

## Introduction

The maintenance of muscle mass as well as muscle strength and functions are essential in various diseases^1,2^. Sarcopenia can increase adverse outcomes, including falls, functional decline, frailty, and mortality^3,4^. In recent years, there have been an increasing number of sarcopenia reports in respiratory diseases, such as chronic obstructive pulmonary diseases (COPD)^5–7^. Furthermore, it has been reported in interstitial lung diseases (ILD)^8–10^; thus, preventing muscle weakness is one of the important strategies for the management of patients with ILD^9^.

Generally, skeletal muscles are used for the assessment of sarcopenia. Researchers predominantly measure in general, the cross-sectional area of the pectoralis muscles (PM_CSA_) and erector spinae muscles (ESM_CSA_) by computed tomography (CT) scans in ILD. Previous studies have reported that decreasing ESM_CSA_^11,12^ and PM_CSA_^13,14^ on CT images may be associated with poor prognosis in patients with ILD.

Titin is a giant structural protein that supports the contraction of actin-containing thin filaments and myosin-containing thick filaments. Titin gets fragmented to form urinary titin N-terminal fragment, and is excreted in the urine^15^. The N-terminal fragments of titin in urine (urinary N-titin/Cr) is a novel biomarker reflecting muscle damage, and has recently attracted considerable attention^16^. The relationship between urinary N-titin/Cr and physical dysfunction has been reported in various clinical populations^17,18^.

The importance of maintaining muscle mass in patients with ILD has been recognized in clinical settings; nevertheless, researchers have not clarified the long-term and specific impact of muscle mass maintenance using biomarkers in patients with ILD. Furthermore, they have not investigated urinary N-titin/Cr in patients with ILD. We hypothesized that urinary N-titin/Cr could be a useful biomarker and predict the loss of muscle mass in the future. Therefore, this study aimed to examine urinary N-titin/Cr as a biomarker reflecting muscle damages in patients with ILD.

## Methods

### Study design

This retrospective observational study enrolled patients with ILD from 2020 to 2021. The study protocol conformed to the ethical guidelines of the 1975 Declaration of Helsinki as reflected in prior approval by the Ethics Committee of Nagasaki University Hospital (approval number: 21051717). The necessary information was obtained from medical charts. We obtained informed consent in the form of an opt-out option on the website; patients who were uninterested in the study were excluded.

### Participants

We recruited patients with ILD, including idiopathic interstitial pneumonias (IIPs), connective tissue disease-associated interstitial pneumonia, and hypersensitivity pneumonitis at the Department of Respiratory Medicine, Nagasaki University Hospital. The diagnostic criteria for IIPs and hypersensitivity pneumonitis were consistent with the International Consensus Statement^19,20^. The inclusion criteria were as follows: under the care of a respiratory physician, were able to walk, and clinically stable without changes in medications for at least 4 weeks before enrollment. The exclusion criteria were as follows: comorbid conditions affecting exercise performance (e.g., musculoskeletal or neurological disorders), severe cognitive impairment, pregnancy, recent thoracic surgery, and active cancer treatment.

### Measurements

#### Urinary N-titin/Cr

Urinary N-titin/Cr was measured as a biomarker reflecting muscle damages. Urine samples were frozen at − 80 °C following collection. We measured urinary N-titin/Cr using an enzyme-linked immunosorbent assay (ELISA) kit (#27900 Human Titin N-Fragment Assay Kit; Immuno-Biological Laboratories, Fujioka, Japan). The value of titin N-fragment concentration was corrected by that of urine creatinine to avoid the effects of concentrated or diluted urine, using the following creatinine ratio: (urinary N-titin/Cr; pmol/mg/dL) = Titin N-fragment (pmol/L)/creatinine (mg/dL)^18,21^.

#### Cross-sectional area of muscle mass

Thoracic CT was performed for the evaluation of muscle mass^11^. CT was performed with 1 mm to 5 mm-thick samples at slice intervals of 1 mm to 5 mm. We analyzed single-slice axial CT images captured at the sum of the cross-sectional areas of the major and minor pectoralis muscles at the top of the aortic arch as the pectoralis muscles areas. By contrast, muscle areas at the 12th thoracic vertebra were measured as the erector spinae to assess the muscle mass. Muscle areas at the aortic arch and Th12 level were semi-automatically defined using SYNAPSE VINCENT™ software (Fujifilm Medical Co., Ltd., Tokyo, Japan), and the muscle area was quantified based on the CT hounsfield unit (HU) range, i.e., − 29 HU to + 150 HU. W assessed the change in muscle mass following 6 months and 1 year from the first evaluation. The measurements were performed five times to confirm the accuracy of CT measurement; the average value of five measurements was recorded.

### ILD-GAP model

We developed the ILD-gender, age, and lung physiology (ILD-GAP) model by adding the ILD subtype variable to the original GAP model^22^. The two lung physiology variables in this model included the percentage of forced vital capacity (%FVC) and the percentage of diffusion capacity for carbon monoxide (%DLco). Points were assigned for each variable to obtain a total point score (range: 0–8). We obtained demographic and clinical information, including physical function, the biochemistry of blood, and pulmonary function test results from medical charts.

### Dyspnea

We assessed dyspnea in daily activities using the modified Medical Research Council (mMRC) dyspnea scale, comprising five statements that reflected perceived breathlessness, each corresponding to a dyspnea severity grade^23^ defined as follows: grade 0, not troubled by breathlessness, except during strenuous exercise; grade 1, short of breath while hurrying or walking up a slight hill; grade 2, walks slower than contemporaries on level ground owing to breathlessness or has to stop for breath while walking at own pace; grade 3, stops for breath after walking about 100 m or after few minutes on the level ground; and grade 4, considerable breathless to leave the house or breathless while dressing/undressing.

### Pulmonary function tests and arterial blood gas analyses

Pulmonary function test measures included the FVC, forced expiratory volume in one second, and DLco. The adopted measurements are expressed as percentages of the predicted values^24^. Furthermore, we measured the arterial partial pressures for oxygen and carbon dioxide within the arterial blood sample at rest. Changes in the pulmonary function test were assessed following 6 months and 1 year from the first evaluation.

### Statistical analyses

Baseline characteristics are summarized with frequencies and percentages for categorical data, in contrast to median and interquartile ranges for continuous data. We used the Spearman's rank coefficient of correlation to examine the relationships among urinary N-titin/Cr, patient background, changes in the cross-sectional area of muscle mass, and baseline characteristics. With regard to urinary N-titin/Cr, we used the upper left corner coordinate point of the receiver operating characteristic (ROC) curve to determine the optimal cut-off level for the discrimination between the greater-than-median and smaller-than-median reduction groups following 1 year of the PM_CSA_ and ESM_CSA_. P-values < 0.05 were considered statistically significant. Overall survival was calculated using the Kaplan–Meier method and compared using the log-rank test. Overall survival was measured from the time of evaluation to death or the last follow-up. All statistical analyses were complete case analyses and performed using JMP 15.0 software (SAS Institute Japan, Tokyo, Japan).

## Results

### Patient characteristics

We enrolled 241 patients with ILD from 2020 to 2021; 166 patients without collecting urine, and 7 patients without sufficient data were excluded from our study. Table 1 summarizes the baseline characteristics of 68 patients. Their median age was approximately 70 years. The ILD diagnoses included IIPs [IPF (n = 27), idiopathic non-specific interstitial pneumonia (n = 2), cryptogenic organizing pneumonia (n = 6), and unclassifiable (n = 16)], connective tissue disease-associated interstitial pneumonia (n = 10), and fibrotic hypersensitivity pneumonitis (n = 7). Thirty-five patients (51.5%) had a history of smoking. Six (8.8%) and four patients (5.9%) were treated with corticosteroids and antifibrotic drugs, respectively. The duration from diagnosis was approximately 1 month to 2 months. The mMRC dyspnea scale was less than grade 1 as mild symptom. Both the PM_CSA_ and ESM_CSA_ were decreased following 6 months and 1 year from the first evaluation. There was no significant difference in urinary N-titin/Cr between patients with or without a history of smoking (median, 9.8 vs 8.6 pmol/mg/dL: p = 0.782).

**Table 1:** Patient characteristics.

	Overall (n = 68)
Age, years	70.0 (64.0 to 77.0)
Sex male, %	39 (57.4)
BMI, kg/m^2^	23.1 (20.4 to 26.2)
Diagnosis, IIPs/other ILDs	49/17
The duration from diagnosis, months	1 (1 to 2)
History of smoking, %	35 (51.5)
Corticosteroids, %	6 (8.8)
Antifibrotic drugs, %	4 (5.9)
Long-term oxygen therapy, %	3 (4.4)
ILD-GAP score, point	2 (1 to 3)
mMRC dyspnea scale, grade	1 (1 to 2)
PaCO_2_ at rest, mmHg	39.2 (36.0 to 42.0)
PaO_2_ at rest, mmHg	79.6 (72.9 to 94.1)
Pulmonary function test at baseline	
FVC, %	84.5 (72.8 to 100.0)
FEV_1_, %pred	89.4 (75.1 to 102.1)
FEV_1_/FVC, %	80.5 (72.7 to 86.2)
DLco, %	69.4 (50.0 to 88.9)
KL-6, U/ml	715.0 (481.5 to 1185.0)
Urinary N-titin/Cr, pmol/mg/dL	7.0 (4.7 to 10.8)
PM_CSA_, cm^2^	30.8 (22.3 to 36.7)
ESM_CSA_, cm^2^	33.4 (25.5 to 41.2)

Values are presented as median (interquartile range) or numbers (percentage).

*BMI* body mass index, *DLco* diffusion capacity for carbon monoxide, *ESM*_*CSA*_ cross-sectional area of erector spinae muscles, *%FEV1* percentage of forced expiratory volume in 1 s, *FVC* forced vital capacity, *IIPs* idiopathic interstitial pneumonias, *ILD-GAP* interstitial lung disease-gender, age, lung physiology score, *KL-6* Krebs von den Lungen-6, *mMRC* modified Medical Research Council, *PaCO*_*2*_ partial pressure of carbon dioxide, *PaO*_*2*_ partial pressure of oxygen, *PM*_*CSA*_ cross-sectional area of pectoralis muscles, *%pred* percent predicted, *%VC* percentage of volume capacity, *urinary N-titin/Cr* urinary N-terminal titin fragment/creatinine.

### Relationships between urinary N-titin/Cr and the cross-sectional area of muscle mass

**Figure 1:**
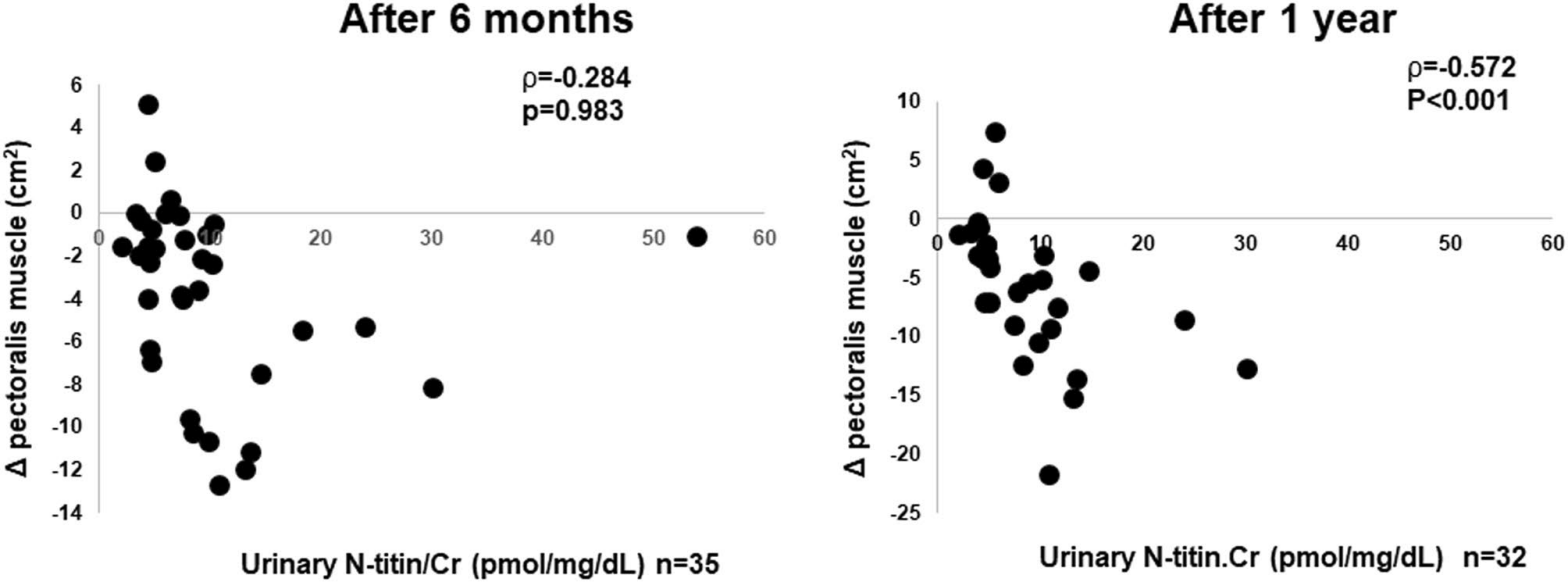
Correlations between urinary N-titin/Cr and changes in the cross-sectional area of the pectoralis muscles in patients with interstitial lung diseases. *Urinary N-titin/Cr* urinary N-terminal titin fragment /creatinine.

**Figure 2:**
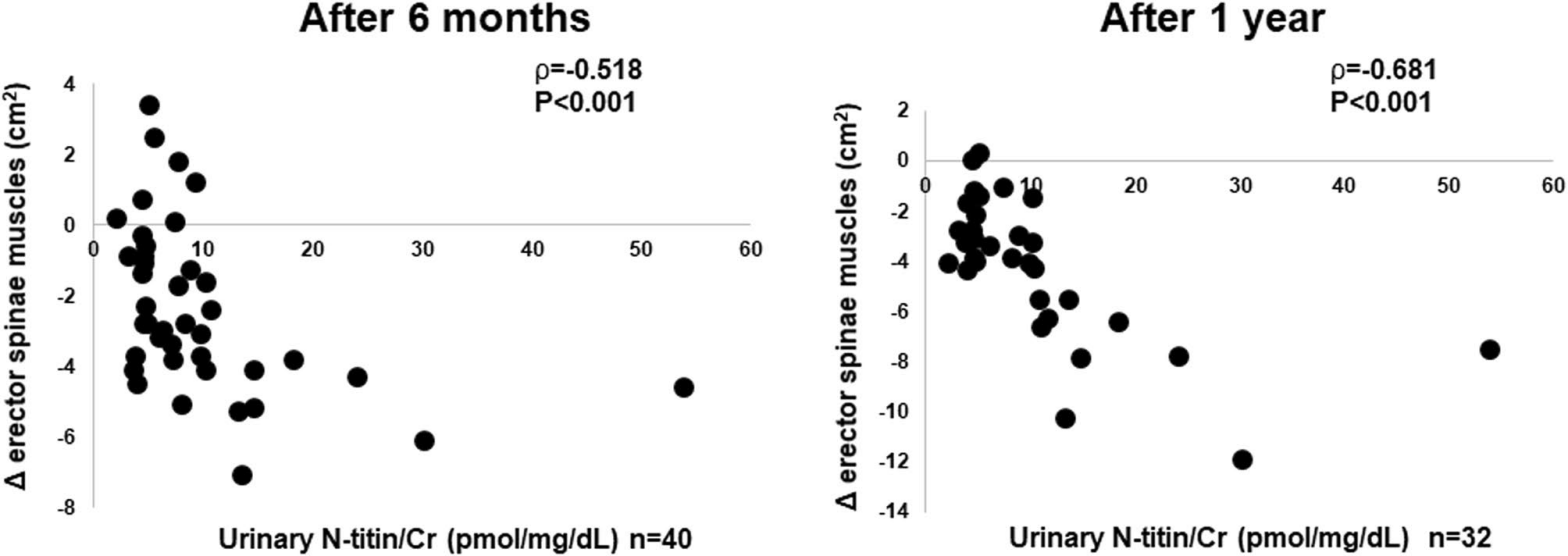
Correlations between urinary N-titin/Cr and changes in the cross-sectional area of the erector spinae muscles in patients with interstitial lung diseases. *Urinary N-titin/Cr* urinary N-terminal titin fragment /creatinine.

Figure 1 depicts the associations between urinary N-titin/Cr and changes in the PM_CSA_. We observed significant negative correlations between urinary N-titin/Cr and changes in the PM_CSA_ after 1 year (ρ = −0.572, p < 0.001) but not after 6 months (ρ = −0.284, p = 0.983). Urinary N-titin/Cr was negatively correlated changes in the ESM_CSA_ after 6 months (ρ = −0.518, p < 0.001) and 1 year (ρ = −0.681, p < 0.001) (Fig. 2). There was no significant correlation between changes in the %FVC and PM_CSA_ after 6 months (ρ = 0.187, p = 0.351) and 1 year (ρ = −0.180, p = 0.368) and the ESM_CSA_ after 6 months (ρ = 0.037, p = 0.855) and 1 year (ρ = 0.272, p = 0.169, data not shown). Urinary N-titin/Cr was not significantly correlated with other background parameters (Table 2).

**Table 2:** Spearman's rank correlation coefficient between urinary N-titin/Cr and baseline characteristics.

	ρ (95%CI)	p-value
Age, years	−0.014 (−0.252 to 0.225)	0.934
BMI, kg/m^2^	0.023 (−0.222 to 0.265)	0.896
The duration from diagnosis, months	0.055 (−0.186 to 0.290)	0.934
ILD-GAP score, point	−0.016 (−0.285 to 0.256)	0.622
mMRC dyspnea scale, grade	0.053 (−0.199 to 0.299)	0.763
PaCO_2_ at rest, mmHg	−0.160 (−0.419 to 0.124)	0.359
PaO_2_ at rest, mmHg	0.241 (−0.040 to 0.487)	0.163
FVC, %	−0.086 (−0.346 to 0.186)	0.624
FEV_1_, %pred	−0.117 (−0.373 to 0.156)	0.505
FEV_1_/FVC, %	0.022 (−0.248 to 0.288)	0.902
DLco, %	−0.022 (−0.286 to 0.244)	0.899
KL-6, U/ml	−0.086 (−0.349 to 0.188)	0.622
PM_CSA_, cm^2^	0.042 (−0.199 to 0.278)	0.735
ESM_CSA_, cm^2^	0.222 (−0.018 to 0.437)	0.069

*BMI* body mass index, *95%CI* 95% confidence interval, *DLco* diffusion capacity for carbon monoxide, *ESM*_*CSA*_ cross-sectional area of erector spinae muscles, *%FEV1* percentage of forced expiratory volume in 1 s, *FVC* forced vital capacity, *IIPs* idiopathic interstitial pneumonias, *ILD-GAP* interstitial lung disease-gender, age, lung physiology score, *KL-6* Krebs von den Lungen-6, *mMRC* modified Medical Research Council, *PaCO*_*2*_ partial pressure of carbon dioxide, *PaO*_*2*_ partial pressure of oxygen, *PM*_*CSA*_ cross-sectional area of pectoralis muscles, *%pred* percent predicted, *%VC* percentage of volume capacity, *urinary N-titin/Cr* urinary N-terminal titin fragment/creatinine.

**Figure 3:**
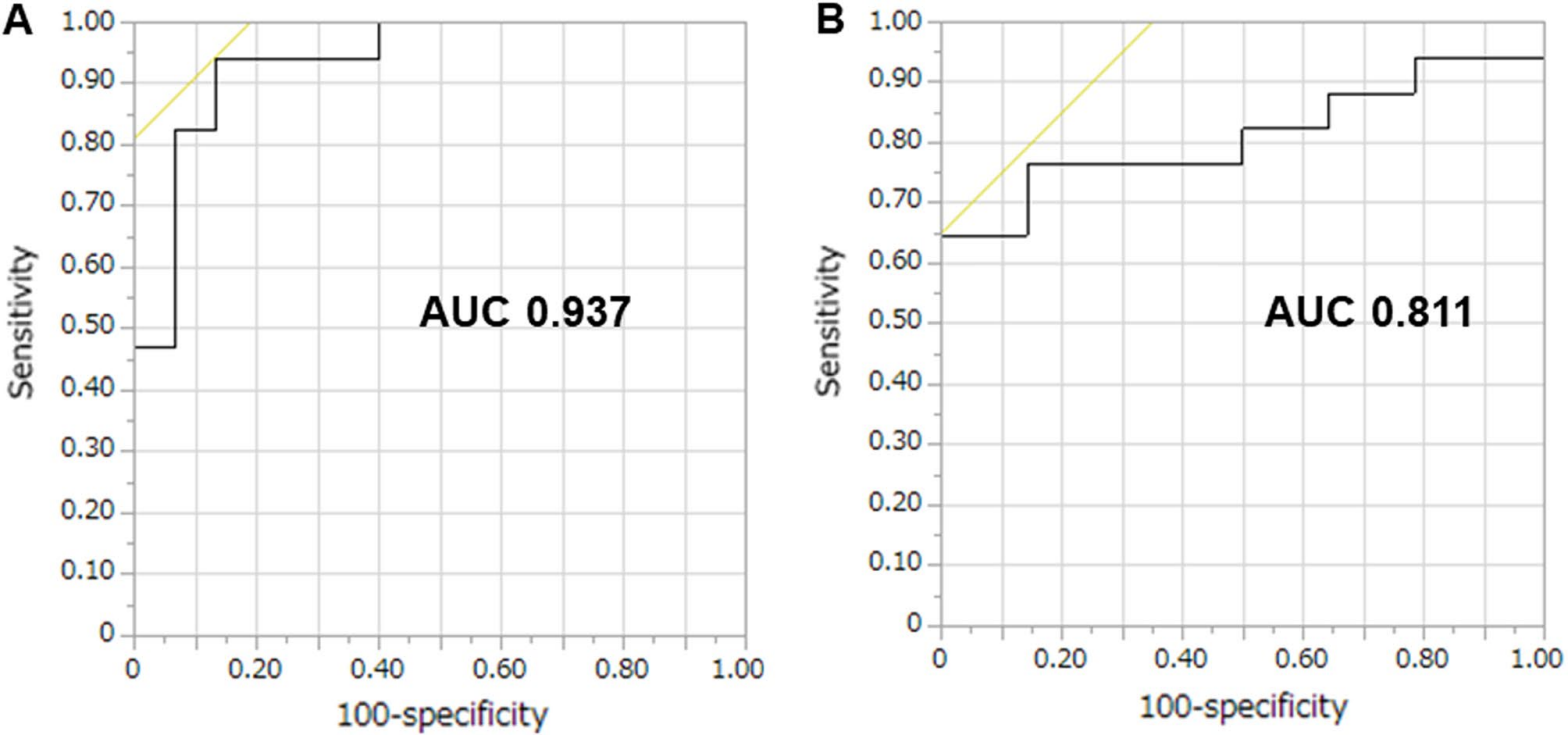
Receiver operating characteristic curves for the cut-off point of urinary N-titin/Cr in patients with interstitial lung diseases. (**A**) AUC in the PM_CSA_ and (**B**) AUC in the ESM_CSA_. *AUC* area under the receiver operating characteristic curve.

Subsequently, we evaluated the sensitivity and specificity of urinary N-titin/Cr levels to distinguish the greater-than-median and smaller-than-median reduction in the PM_CSA_ and ESM_CSA_ after 1 year based on ROC curves (Fig. 3). The areas under the curve for urinary N-titin/Cr on the PM_CSA_ and ESM_CSA_ were 0.937 and 0.811, respectively. Based on the ROC curve, the cut-off level for urinary N-titin/Cr was 5.2 pmol/mg/dL and 10.4 pmol/mg/dL on the PM_CSA_ (sensitivity, 94.1%; specificity, 86.7%) and ESM_CSA_ (sensitivity, 64.7%; specificity, 100.0%), respectively. A total of six (8.8%) deaths occurred during the investigative period. At 2 years after the evaluation of urine N-titin/Cr, no one died in the greater-than-median reduction in PM_CSA_ group and three of 17 patients (17.6%) died in the smaller-than-median reduction in PM_CSA_ group (log-rank: p = 0.08). Moreover, two of 14 patients (14.3%) in the greater-than-median reduction in ESM_CSA_ group and two of 17 (11.8%) in the smaller-than-median reduction in ESM_CSA_ group died during the same period (log-rank: p = 0.84).

## Discussion

The primary findings of this study were as follows: (1) the median urinary N-titin/Cr was 7.0 pmol/mg/dL in patients with ILD; (2) significant negative correlations were observed between urinary N-titin/Cr and changes in the PM_CSA_ and ESM_CSA_. This is the first report investigating urinary N-titin/Cr in patients with ILD.

The median urinary N-titin/Cr was 7.0 pmol/mg/dL and 2.0 pmol/mg/dL in patients with ILD and healthy adult volunteers^25^. Titin, a giant sarcomere protein, is cleaved by calpain-3 in damaged muscles, and the resulting N-terminal fragments are excreted into the urine via glomerular filtration. Maruyama et al.^25^ established a highly sensitive, simple, and non-invasive sandwich ELISA system for evaluating muscle injury. Thus, research on muscle damage using the ELISA system has been active in recent years.

Although the elevation of urinary N-titin/Cr did not reach at a higher level as acute inflammatory condition such as nonsurgical critically ill patients on 7 day of intensive care unit admission (49.3 pmol/mg/dL)^16^ and patients who underwent cardiac surgery on the day of operation (43.3 pmol/mg/dL)^26^, that was at a similar level as chronic diseases such nonalcoholic fatty liver disease (NAFLD) and dilated cardiomyopathy^18^. They have an important clinical implication on the elevation of urinary N-titin/Cr: the urinary N-titin/Cr in patients with NAFLD reflected skeletal muscle deterioration and functional decline, and was associated with hepatic pathological conditions. Urinary N-titin/Cr in patients with dilated cardiomyopathy acted as a predictor of cardiac and all-cause mortality^27^.

Urinary N-titin/Cr in patients with ILD was not associated with their baseline information (including KL-6 level), disease severity (assessed using the ILD-GAP score), lung function, PM_CSA_, or ESM_CSA_ during evaluation (Table 2). This may be explained by the failure of urinary N-titin/Cr, a marker of sarcomere damage, to directly reflect muscle mass rather muscle atrophy^28^.

Interestingly, the reduction in the PM_CSA_ after 1 year and ESM_CSA_ after 6 months and 1 year were significantly associated with urinary N-titin/Cr. The ESM_CSA_ was reportedly correlated with the physiological parameters, symptoms, and disease prognosis^29^ in patients with COPD, besides being an independent mortality risk factor in elderly patients with pneumonia^30^. In addition, the ESM_CSA_ was a poor prognostic factor in patients with ILD^9,11,12^. Particularly, Suzuki et al. reported that low ESM_CSA_ was an independent prognostic factor associated with worse survival rates even in patients with IPF receiving antifibrotic therapy. In addition, it is essential to prevent sarcopenia for IPF management^9^. Furthermore, the PM_CSA_ was correlated with mortality in ever smokers^31^ and the total energy expenditure and physical activity level in patients with COPD^32^. In patients with ILD, lower PM_CSA_ was associated with the skeletal mass index, quadriceps isometric maximal voluntary contraction, and the indicators of ILD severity^8,14,33^, besides being a poor prognostic factor^14^.

Recently, the loss of skeletal muscle mass represented by sarcopenia has been considered important for evaluating the predictors of mortality in patients with various diseases, including ILD. However, it is difficult to predict the muscle mass in patients with ILD in the future. Considering significant negative correlations between urinary N-titin/Cr and muscle mass, our findings suggested that urinary N-titin/Cr in chronic patients with ILD could predict the future reduction in the PM_CSA_ and ESM_CSA_ associated with the skeletal muscle mass, strength, and prognosis in patients with ILD. In our study, the amount of reduction in the PM_CSA_ and ESM_CSA_ was not sufficient to predict mortality. In addition, because the number of patients who died during the investigation period was small, multiple comparisons would reduce statistical power to detect significance and further consideration was not possible.

Moreover, our cut-off values of urinary N-titin/Cr (5.2 pmol/mg/dL and 10.4 pmol/mg/dL) based on the ROC curve analysis could deduce the greater reduction of the PM_CSA_ and ESM_CSA_ compared with the median value after 1 year. Therefore, upon anticipating the reduction in muscle mass by simple measurements of urinary N-titin/Cr levels, we could implement an aggressive intervention, such as pulmonary rehabilitation program and nutritional support, in patients with ILD. This in turn may improve the disease prognosis.

This study had several limitations. First, the sample size was small, and we did not consider missing values in this study. Second, the study was based on a retrospective design. Third, it was conducted at a single institute. Lastly, most of our patients were mild cases (ILD-GAP score; 1 [0–2]). Therefore, our results were subject to selection bias and should be interpreted with caution. Larger multicenter studies are required to consider the detailed efficacy of urinary N-titin/Cr as a biomarker reflecting muscle damages. Moreover, we did not evaluate all patients for their physical function and performance (e.g., handgrip and quadriceps force and 6-min walking test); therefore, we could not investigate the relationship between physical performance and urinary N-titin/Cr. Despite the limitations, the strength of our study was that it clarified the usefulness of urinary N-titin/Cr in patients with ILD. Our findings will facilitate the development of novel prevention strategies for patients with ILD, thereby reducing the long-term effects of physical dysfunction and health care costs.

In conclusion, urinary N-titin/Cr of patients with ILD was high value similar other diseases, and urinary N-titin/Cr may be predictive of the skeletal muscle loss in the future. Particularly, clinicians should adopt earlier countermeasures, such as pulmonary rehabilitation, to maintain the muscle mass in patients with ILD and abnormalities in urinary N-titin/Cr level. This necessitates verifying the effectiveness of urinary N-titin/Cr in internal and external validity.

## Data Availability

The datasets generated and analyzed during the current study are not publicly available due to data protection policies but are available from the corresponding author on reasonable request.

## Abbreviations

BMI: Body mass index
DLco: Diffusion capacity for carbon monoxide
%FEV1: Percentage of forced expiratory volume in one second
FEV1/FVC: Forced expiratory volume in one second/forced vital capacity
IIPs: Idiopathic interstitial pneumonias
ILD: Interstitial lung disease
ILD-GAP model: Interstitial lung disease-gender, age, lung physiology
IPF: Idiopathic pulmonary fibrosis
mMRC dyspnea: Modified Medical Research Council dyspnea
PaCO_2_: Partial pressure of carbon dioxide
PaO_2_: Partial pressure of oxygen
%pred: Percent predicted
%FVC: Percentage of forced vital capacity
Urinary N-titin/Cr: Urinary N-terminal titin fragment/creatinine

## References

[1] Marklund S, Bui KL, Nyberg A (2019). Measuring and monitoring skeletal muscle function in COPD: Current perspectives. Int. J. Chron. Obstruct. Pulmon. Dis..

[2] Tyrovolas S (2020). Skeletal muscle mass in relation to 10 year cardiovascular disease incidence among middle aged and older adults: The Attica study. J. Epidemiol. Commun. Health.

[3] Cruz-Jentoft AJ, Sayer AA (2019). Sarcopenia. Lancet.

[4] Woo J, Leung J, Morley JE (2015). Defining sarcopenia in terms of incident adverse outcomes. J. Am. Med. Dir. Assoc..

[5] Benz E (2019). Sarcopenia in COPD: A systematic review and meta-analysis. Eur. Respir. Rev..

[6] Sepúlveda-Loyola W (2020). Diagnosis, prevalence, and clinical impact of sarcopenia in COPD: A systematic review and meta-analysis. J. Cachexia Sarcopenia Muscle.

[7] Bone AE, Hepgul N, Kon S, Maddocks M (2017). Sarcopenia and frailty in chronic respiratory disease. Chron. Respir. Dis..

[8] Ebihara K (2021). Appendicular skeletal muscle mass correlates with patient-reported outcomes and physical performance in patients with idiopathic pulmonary fibrosis. Tohoku J. Exp. Med..

[9] Suzuki Y (2021). Cause of mortality and sarcopenia in patients with idiopathic pulmonary fibrosis receiving antifibrotic therapy. Respirology.

[10] Hanada M (2022). A comparative study of the sarcopenia screening in older patients with interstitial lung disease. BMC Pulm. Med..

[11] Awano N (2020). Quantitative computed tomography measures of skeletal muscle mass in patients with idiopathic pulmonary fibrosis according to a multidisciplinary discussion diagnosis: A retrospective nationwide study in Japan. Respir. Investig..

[12] Suzuki Y (2018). Distinct profile and prognostic impact of body composition changes in idiopathic pulmonary fibrosis and idiopathic pleuroparenchymal fibroelastosis. Sci. Rep..

[13] Moon SW (2019). Thoracic skeletal muscle quantification: low muscle mass is related with worse prognosis in idiopathic pulmonary fibrosis patients. Respir. Res..

[14] Molgat-Seon Y (2021). Pectoralis muscle area and its association with indices of disease severity in interstitial lung disease. Respir. Med..

[15] Nakanishi N (2021). Urinary titin N-fragment as a biomarker of muscle atrophy, Intensive Care Unit-acquired weakness, and possible application for post-intensive care syndrome. J. Clin. Med..

[16] Nakanishi N (2020). Urinary titin is a novel biomarker for muscle atrophy in nonsurgical critically ill patients: A two-center, prospective observational study. Crit. Care Med..

[17] Awano H (2018). Diagnostic and clinical significance of the titin fragment in urine of Duchenne muscular dystrophy patients. Clin. Chim. Acta.

[18] Oshida N (2019). Urinary levels of titin-N fragment, a skeletal muscle damage marker, are increased in subjects with nonalcoholic fatty liver disease. Sci. Rep..

[19] Raghu G (2018). Diagnosis of idiopathic pulmonary fibrosis. An official ATS/ERS/JRS/ALAT clinical practice guideline. Am. J. Respir. Crit. Care Med..

[20] Raghu G (2020). Diagnosis of hypersensitivity pneumonitis in adults. An official ATS/JRS/ALAT clinical practice guideline. Am. J. Respir. Crit. Care Med..

[21] Nakano H (2021). Urine titin N-fragment as a biomarker of muscle injury for critical illness myopathy. Am. J. Respir. Crit. Care Med..

[22] Ryerson CJ (2014). Predicting survival across chronic interstitial lung disease: The ILD-GAP model. Chest.

[23] Bestall JC (1999). Usefulness of the Medical Research Council (MRC) dyspnoea scale as a measure of disability in patients with chronic obstructive pulmonary disease. Thorax.

[24] Hanamoto S, Ohsuji T, Tsuyuguchi I, Kawabata S, Kimura K (1992). Prediction formulas for pulmonary function tests expressed in linear and exponential form for healthy Japanese adults. Nihon Kyobu Shikkan Gakkai Zasshi.

[25] Maruyama N (2016). Establishment of a highly sensitive sandwich ELISA for the N-terminal fragment of titin in urine. Sci. Rep..

[26] Tanihata J, Nishioka N, Inoue T, Bando K, Minamisawa S (2019). Urinary titin is increased in patients after cardiac surgery. Front. Cardiovasc. Med..

[27] Yoshihisa A (2018). Usefulness of urinary N-terminal fragment of titin to predict mortality in dilated cardiomyopathy. Am. J. Cardiol..

[28] Matsuo M, Awano H, Maruyama N, Nishio H (2019). Titin fragment in urine: A noninvasive biomarker of muscle degradation. Adv. Clin. Chem..

[29] Tanimura K (2016). Quantitative assessment of erector spinae muscles in patients with chronic obstructive pulmonary disease. Novel chest computed tomography-derived index for prognosis. Ann. Am. Thorac. Soc..

[30] Yoshikawa H (2021). Quantitative assessment of erector spinae muscles and prognosis in elderly patients with pneumonia. Sci. Rep..

[31] Mason SE (2022). Longitudinal association between muscle loss and mortality in ever smokers. Chest.

[32] Shirahata T (2021). The product of trunk muscle area and density on the CT image is a good indicator of energy expenditure in patients with or at risk for COPD. Respir. Res..

[33] Pietro KM (2017). Relationship of pectoralis muscle area and skeletal muscle strength with exercise tolerance and dyspnea in interstitial lung disease. Sarcoidosis Vasc. Diffuse Lung Dis..

